# Supplementary material for: Involvement of Mitochondrial Mechanisms and Cyclooxygenase-2 Activation in the Effect of Desethylamiodarone on 4T1 Triple-Negative Breast Cancer Line
Source: Int J Mol Sci. 2022 Jan 28;23(3):1544. doi: 10.3390/ijms23031544 (PMC8836269; doi:10.3390/ijms23031544)
Supplement: Supplementary file 1 [file ijms-23-01544-s001.zip › ijms-1511504 - supplementary for publication corrected 1-27.pdf]

Supplementary Materials:

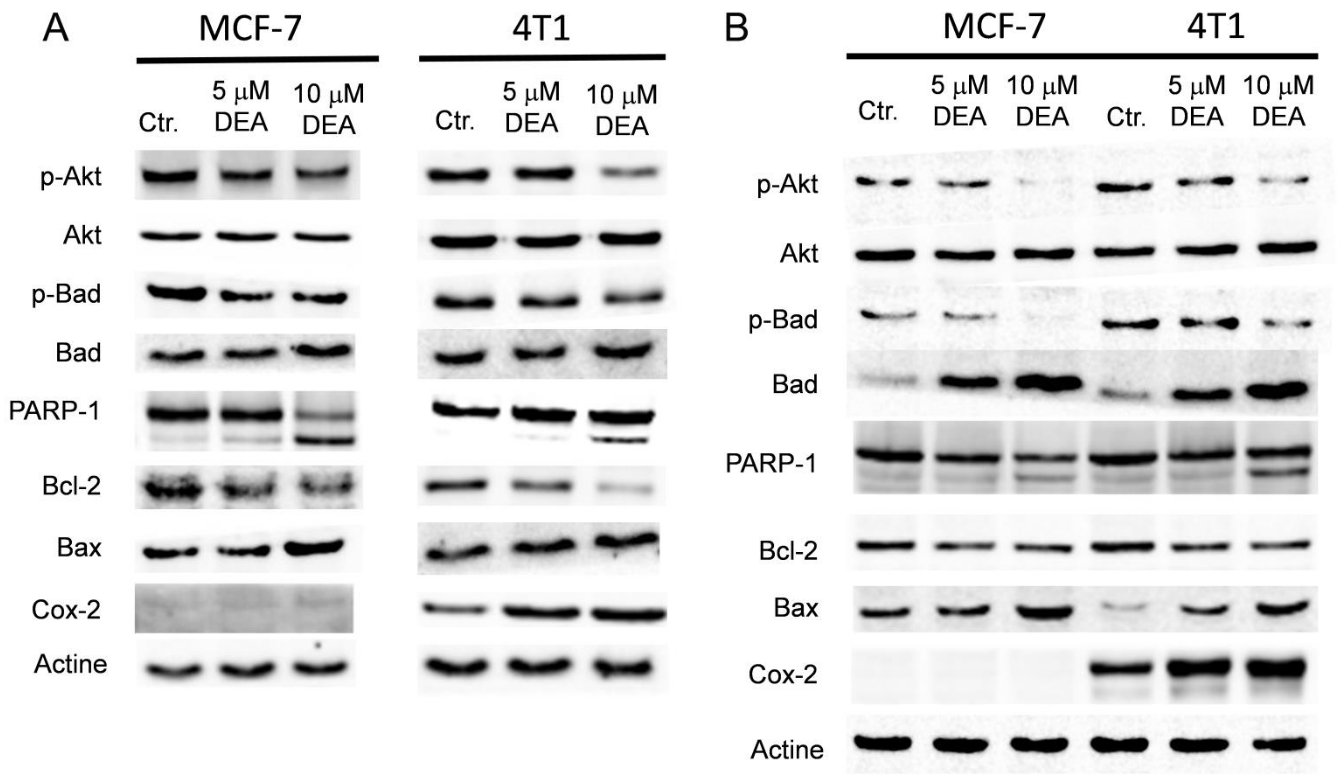

Supplementary Figure S1: Effect of DEA on steady-state level of regulator- and marker proteins of the cell death process in the BC cell lines. We evaluated the steady-state levels of p-Akt, Akt, p-Bad, Bad, Bcl-2, Bax, PARP-1 and COX-2 in the MCF-7 and 4T1 cells treated by 0, 5 and 10 μM DEA by immunoblot analysis. Results are presented as representative immunoblots of three independent experiments (A) separate from those presented in Figure 4. The experiment was repeated and the samples were loaded on the same gel for comparative purposes B).

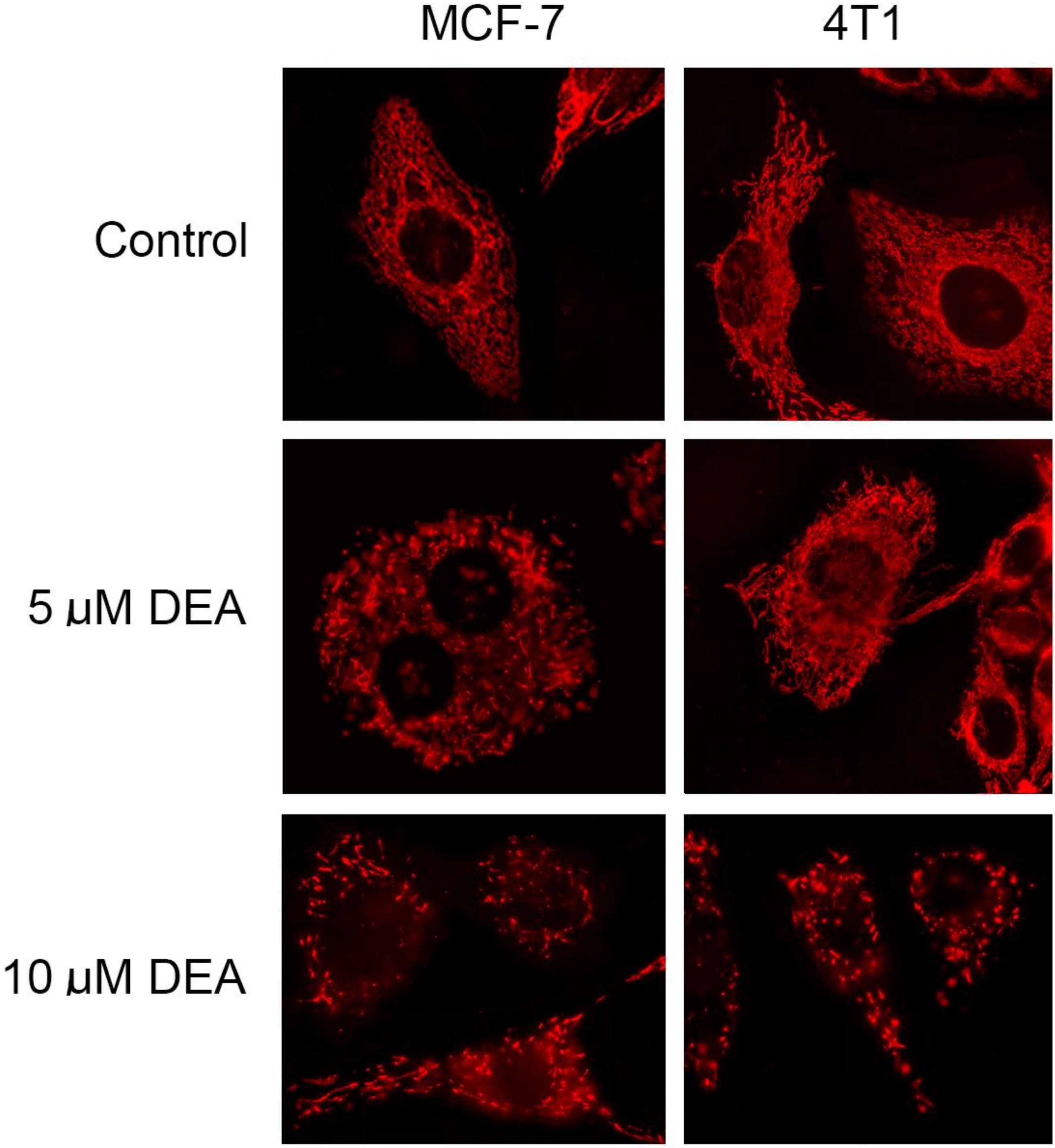

Supplementary Figure S2. Effect of DEA on mitochondrial network dynamics in BC cell lines. MCF-7 and 4T1 cells were treated with 0, 5 or 10 µM DEA for 3 h before loading them with MitoTracker Red dye and taking microscopy images. The data are presented as representative images of three independent experiments separate from those presented in Figure 6.

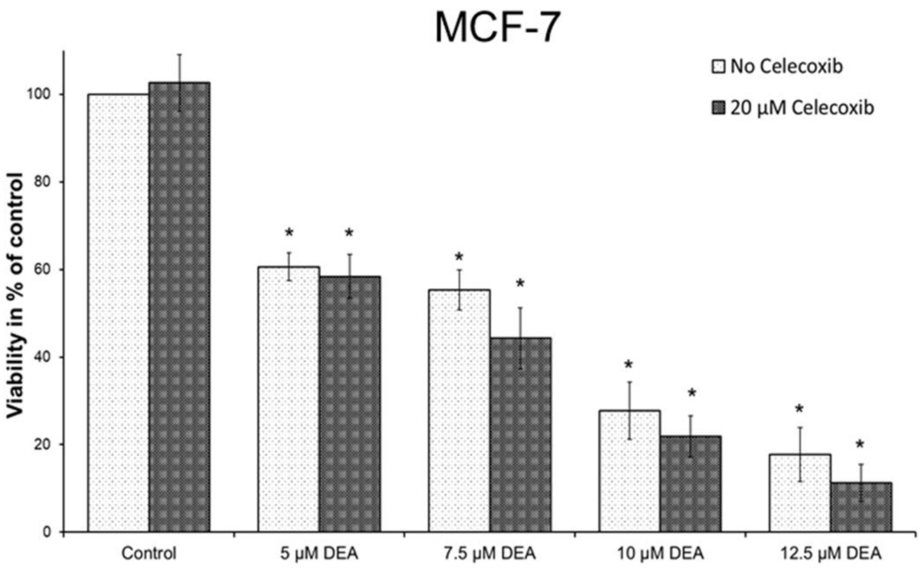

Supplementary Figure S3. We treated MCF-7 cells with 0 to 12.5 µM DEA in the absence (light bars) or presence (dark bars) of 20 µM celecoxib for 24 h. Viabilities were assessed using the SRB assay, and were presented as percent of the untreated control, means ± SD of three independent experiments performed in at least quadruplicates. * significant difference from the untreated control ($p<0.05$); § significant difference from the no celecoxib parallel ($p < 0.05$).
